# Appropriateness of the post-operative rehabilitation of low energy hip fractures in elderly in comparison with the AAOS appropriate use criteria at a level one trauma center

**DOI:** 10.1007/s00590-021-02938-w

**Published:** 2021-03-30

**Authors:** Mohammed Adam, Eslam Alkaramany, Abduljabbar Alhamoud, Jawad Derbas, Abdullah Murshid, Ghalib Ahmed Alhaneedi

**Affiliations:** Orthopedics Department, Hamad General Hospital, Hamad Medical Corporation, PO Box 3050, Doha, Qatar

**Keywords:** American Academy of Orthopedic Surgeons (AAOS), Appropriate Use Criteria, Elderly, Hip fracture, Post-operative rehabilitation, Trauma center

## Abstract

**Purpose:**

This study aimed to assess the appropriateness of the post-operative rehabilitation of low energy hip fractures in the elderly by comparing between the rehabilitations actually provided at level one trauma center and the AAOS Appropriate Use Criteria (AUC) recommendations.

**Methods:**

A retrospective review of the medical charts of all patients who underwent surgery for hip fractures followed by post-operative rehabilitation between October 2016 and May 2018. The age, gender, fracture types, four AUC variables including; the surgical approach, pre-operative mobility/functional status, cognitive impairment, and post-operative delirium, and types of post-operative rehabilitation received were collected.

The four patient variables were entered into the AUC application to generate the recommended rehabilitation procedures. Afterward, the rate of appropriateness of the treatments and the agreement between the rehabilitations actually provided and the AUC recommendation were measured.

**Results:**

Over the study period, a consecutive series of 101 patients were included. The mean age was 75 years. Most of the patients were males (51.5%). Seventeen scenarios were observed in our patients. The most common scenario were patients with low functional/physical demands (48%), intact cognitive function (91%), non-arthroplasty approach (76%), and no post-operative delirium(97%).

The overall appropriateness rate of the provided rehabilitation treatments for our patients in comparison with AUC recommendation was appropriate in 356 (48.7%) (*P* = .001), maybe appropriate in 19 (3%) (*P* < .001), rarely appropriate in 61 (8.3%) (*P* = .59), and 40% of rehabilitation procedures were not provided (*P* < .001).

The actual treatment was appropriate and in agreement with the AUC recommendations in (100%) of three procedures (Deep venous thrombosis prophylaxis, pain management, and Inpatient Rehabilitation Facility or Skilled Nursing Facility), in (72.2%) of osteoporosis assessment/management, in (63.8%) of outpatient occupational/physical therapy, in (10.2%) of delirium prevention, in (33.3%) of delirium management and in (25%) of home care therapy.

**Conclusions:**

This study demonstrated that there is a remarkable variation in the appropriateness of the various post-operative rehabilitation procedures for elderly hip fracture. Additionally, the AUC application was easy to use and simple for identifying post-operative rehabilitation protocols for elderly hip fractures, hence, we recommend to use it in the trauma clinical practice.

**Level of evidence:** IV

## Introduction

Hip fracture is one of the most common fractures in the Elderly. It is usually caused by low-energy trauma. It is often associated with osteoporosis and other medical co-morbidities that may increase the prevalence of falls [1–3]. Most fractures occur in women older than 65 years, with an estimated worldwide incidence of approximately 1.7 million per year [2, 3].

Elderly patients with hip fractures are at risk for decreased level of mobility, inability to return to prior living circumstances, impaired quality of life, and increased rate of mortality [2–9].

Hip fractures are almost always treated surgically with either internal fixation or arthroplasty depending on the fracture type, age of the patient, fracture site, and pre-injury functional status [10, 11]. The post-operative rehabilitation of hip fractures in the elderly depends on several factors, including fracture type and surgery, the patient-related factors such as pre-operative mobility/functional status, cognitive impairment and post-operative delirium [12]. Post-operative physiotherapy and occupational therapy mainly focus on the function, the mobility of the patient, daily life activities, and independent living [12–14].

To improve the quality of the management of hip fractures in the elderly, the American Academy of Orthopedic Surgeons (AAOS) published clinical practice guidelines in 2014 based on the best available evidence [2, 3].

Subsequently, the AAOS published the Appropriate Use Criteria (AUC) in 2015 for the Post-operative Rehabilitation of Low Energy Hip Fractures in the Elderly (PORHFE) based on the relevant evidence and experts from different fields of musculoskeletal care. The AAOS made the free web-based AUC application widely available to help orthopedic surgeons to select the most appropriate post-operative rehabilitation protocols [15, 16].

Four clinical variables for a specific patient clinical scenario are entered into the AUC application: a surgical approach for arthroplasty (or non-arthroplasty); pre-operative mobility/functional status; cognitive impairment; and post-operative delirium. The application then provides a listing of upto 10 different rehabilitation procedure recommendations. Each procedure is rated into one of three categories: appropriate, may be appropriate, and rarely appropriate [15, 17].

This study aimed to assess the appropriateness of the post-operative rehabilitation of low energy hip fractures in the elderly by comparing between the rehabilitations actually provided at a level one trauma center and the AAOS Appropriate Use Criteria (AUC) recommendations.to determine the rate of appropriateness and agreement with AUC recommendations.

## Methods and materials

### Study design and setting

The Institutional Medical Research Center approved this study with a protocol number (MRC-01-18-072), and informed consent was exempted. Retrospectively, all elderly hip fractures (≥ 60 years) who underwent surgical treatments (fixation or arthroplasty) between October 2016 and May 2018 were identified from the operating theater registry. Our institution is a level 1 trauma center accredited by Joint Commission International (JCI) and Accreditation Council of Graduate Medical Education-International (ACGME-I). Our orthopedic unit includes 14 consultant orthopedic surgeons who manage orthopedic trauma, including hip fractures in elderly patients.

### Eligibility criteria

The eligibility criteria were according to the AUC criteria for post-operative rehabilitation of hip fracture in elderly.

The inclusion criteria were patients who underwent surgery (fixation or arthroplasty) for low-energy isolated elderly hip fractures (≥ 60 years).

Patients with previous surgical intervention, revision surgery, bilateral hip fractures, pathological fractures, and open fractures, poly trauma, and medical contraindications to post-operative rehabilitation therapies were excluded because those patients need special care and multidisciplinary approach which differs from the standard post-operative rehabilitation procedure.

### Data collection

The data for patient age, gender, the surgical approach for arthroplasty or non-arthroplasty, pre-operative mobility/functional status, cognitive impairment, post-operative delirium, and post-operative rehabilitation treatment procedures were collected from the patients’ medical charts and radiographs.

The AUC for post-operative rehabilitation of low energy hip fractures in the elderly requires four patients’ variables to generate appropriateness ratings for ten post-operative rehabilitation procedures. These variables include 1. Surgical approach for arthroplasty, which was retrieved from surgeon operative note and could be either Posterior, Anterior/Anterolateral, or Non-arthroplasty procedure. 2. Pre-operative Mobility/function status graded in four levels from high functioning to non-ambulatory. It was retrieved from admitting physician and physiotherapist notes. 3. Cognitive impairment was retrieved from an emergency physician triage or attending physician assessment notes. It was assessed using Mini-mental state examination (MMSE) and graded into three levels; intact, mild, and moderate or severe. 4. Post-operative delirium was collected from the post-operative attending physician or anesthetist assessment notes.

Thus, the four parameters according to the criteria of the AUC were retrieved by two authors for 101 consecutive patients included in this study and entered into the AUC application to generate the post-operative rehabilitation protocols.

To assess the appropriateness of post-operative rehabilitation, first, the four parameters of each patient were input into the AUC to generate the appropriateness rating of the ten provided rehabilitation procedures for each patient.

**Fig. 1:**
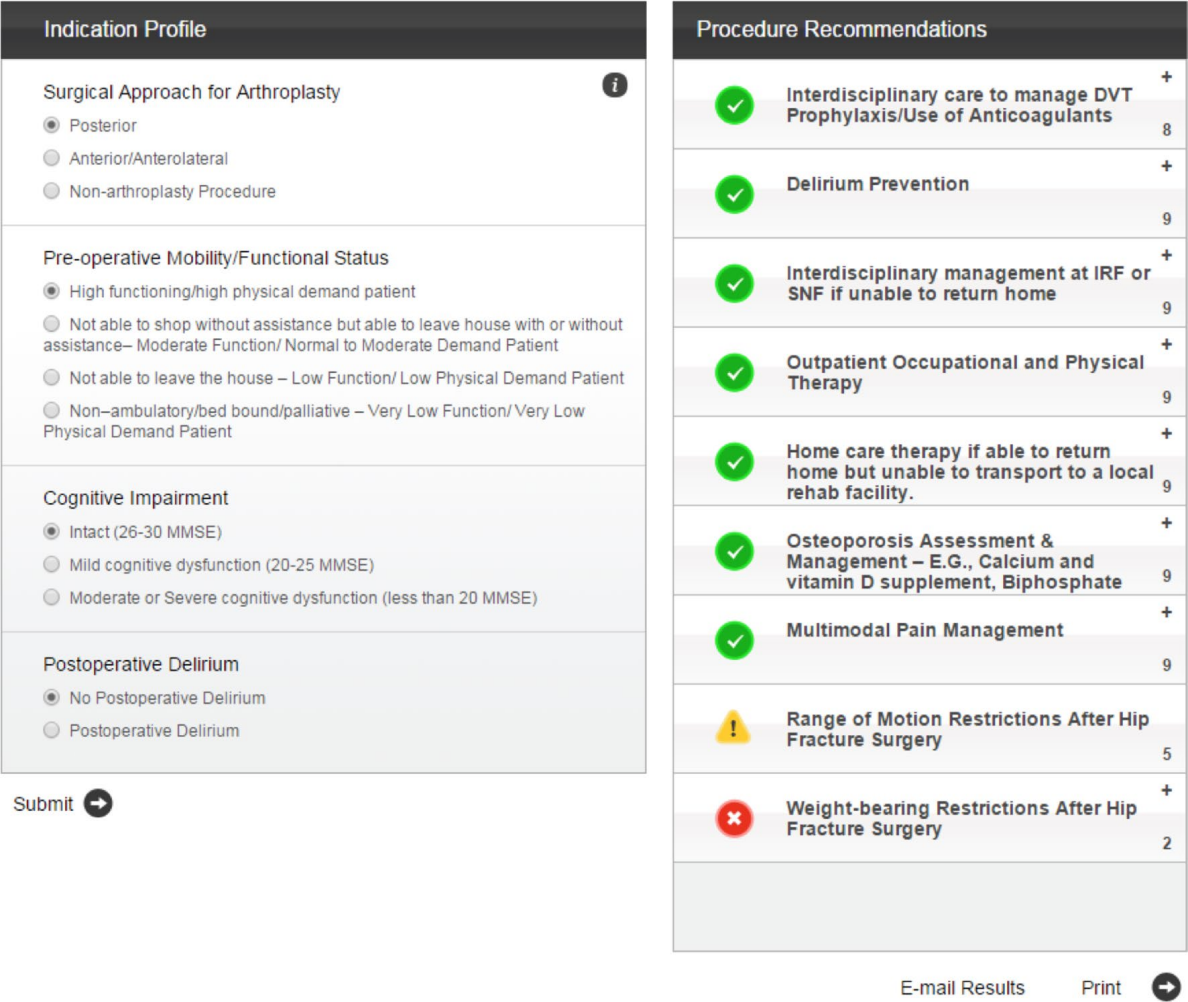
Web-based AUC application screenshot [15]

Each of the ten rehabilitation procedures is rated as appropriate, may be appropriate, or rarely appropriate according to the AUC application (Fig. 1 and Table 1).

**Table 1:** Interpreting the final ratings of appropriate use criteria [15]

Level of appropriateness	Description
Appropriate	Median panel rating between 7 and 9 and no disagreement
Maybe appropriate	Median panel rating between 4 and 6, or
	Median panel rating 1–9 with disagreement
Rarely appropriate	Median panel rating between 1 and 3 and no disagreement
Appropriate treatment is generally acceptable, is a reasonable approach for the indication, and is likely to improve the patients’ health outcomes or survival	
May be Appropriate treatment may be acceptable and may be a reasonable approach for the indication, but with uncertainty implying that more research and/or patient information is needed to further classify the indication	
Rarely an appropriate option for management of patients in this population due to the lack of a clear benefit/risk advantage; rarely an effective option for individual care plans; exceptions should have documentation of the clinical reasons for proceeding with this care option (i.e., procedure is not generally acceptable and is not generally reasonable for the indication)	

Afterward, the appropriateness rate and agreement with the AUC recommendations were then compared between the treatments actually provided and the AUC recommendations.

The AAOS panels made amendments to remove the below treatment options from specific patient scenarios due to clinical irrelevance:

- Delirium management was removed in scenarios with no post-operative delirium.
- Delirium prevention was removed in patients with post-operative delirium.
- Outpatient Rehabilitation was removed in the patient scenarios with the inability to leave the house (low function patient).
- Outpatient Rehabilitation was removed in scenarios with non-ambulatory/bed dependent.
- Interdisciplinary management at Inpatient Rehabilitation or Skilled Nursing Facility (IRF or SNF) was provided only if the patient unable to return home.
- Home care therapy was provided if able to return home but unable to transport to a local rehab facility.
- Thus, according to the above-mentioned amendments; two hundred seventy-eight rehabilitation procedures for 101 patients were removed. Hence, 732 rehabilitation procedures should be provided for our patients.

### Statistical analysis

Descriptive statistics such as means, ranges, ± standard deviation, and frequency were used to describe the continuous variables such as the patient characteristics and patient scenarios. Whereas discrete variables were described as frequency (percentage of total) such as appropriateness rating for each rehabilitation procedure and the agreement of the treatments implemented at our institution with the AUC recommendations. Continuous and discrete variables were analyzed via *T* test and Chi-squared test, respectively. A *p* value < 0.05 was considered statistically significant. All data were analyzed using statistical software (IBM SPSS version 22; SPSS Inc., Chicago, IL).

No sample size calculations were performed before conducting this study because all patients who met the inclusion criteria were included. A post hoc power analysis revealed a power of greater than 80%, which indicated that the sample size was adequate for analysis. The calculation was based on using post-hoc calculator with a one sample proportion test and a 5% level of significant to evaluate the power of existing study to detect a difference by comparing our appropriateness rate (48.7%) of 101 cases versus the AAOS AUC appropriateness rate of 75% that was published in previous literature.

## Results

### Participants

**Fig. 2:**
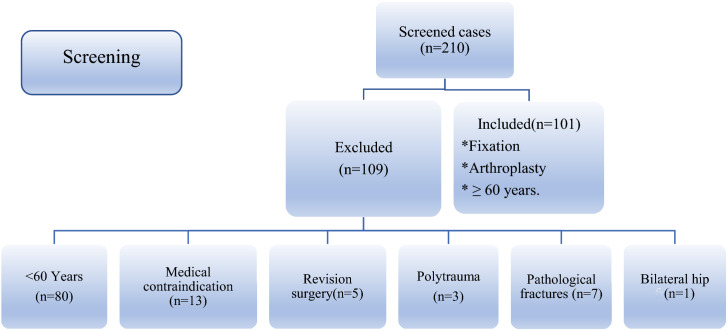
The flowchart summarizing screening, inclusion and exclusion of patients

A total of 210 cases were identified in our theater registry with a diagnosis of hip fractures. One hundred and nine cases were excluded; the reasons for exclusion were < 60 years in 80 patients, medical contraindication to post-operative rehabilitation in 13 cases, revision surgery in five patients, three cases were polytrauma, pathological fractures in seven patients and one patient with bilateral hip fractures. Thus, 101 patients with elderly hip fractures met the inclusion criteria and eligible for analysis (Fig. 2).

### Descriptive data

The mean age was 75.6 years (range 60–96 y), and 51.5% were males. Forty-one case (40.6%) sustained stable intertrochanteric, 23 (22.8%) unstable intertrochanteric fractures, 7 (6.9%) subtrochanteric, 26 (25.8%) displaced neck of femur, 4 (3.9%) nondisplaced neck of femur fractures.

Seventeen out of 72 AUC scenarios were observed in our study. With predominance of low function/low physical demands (48%), intact cognitive function (91%), non-arthroplasty approach (76%) (stable intertrochanteric fracture (40.5%) fixed by DHS (44.5%)) with no post-operative delirium (97%). (Table 2).

**Table 2:** Patients’ variables and characteristics

Patients’ characteristics	Frequency	Percentage
*Surgical approach*		
Posterior	7	6.9%
Anterior/anterolateral	17	17.1%
Non-arthroplasty	77	76%
*Pre-operative mobility/functional status*		
High functioning/ high demand	14	13.8%
Moderate functioning (able to leave house with or without assistance)	35	34.6%
Low functioning (not able to leave house with or without assistance)	48	47.5%
Non-ambulatory (bed dependent)	4	47.5% 3.9%
*Cognitive impairment*		
Intact	92	91.1%
Mild cognitive dysfunction	4	4%
Moderate or severe cognitive dysfunction	5	4.9%
*Post-operative delirium*		
Yes	3	3%
No	98	97%
*Gender*		
Male	52	51.5%
Female	49	48.5%
*Age*		
Mean	75.6 years	
Range	60–96 years	
*Length of hospital stay*		
Mean	12 days	
Range	2–74 days	

### Outcome data

In comparison with AUC recommendation, four hundred thirty-six (60%) rehabilitation procedures were provided, and 296 (40%) of rehabilitation procedures were not provided for our patients (*P* < 0.001).

The overall appropriateness rate of the provided rehabilitation treatments for our patients in comparison with AUC recommendation was appropriate in 356 (48.7%) (*P* = 0.001), maybe appropriate in 19 (3%) (*P* < 0.001), and rarely appropriate in 61 (8.3%) (*P* = 0.59) of rehabilitation procedures.

**Fig. 3:**
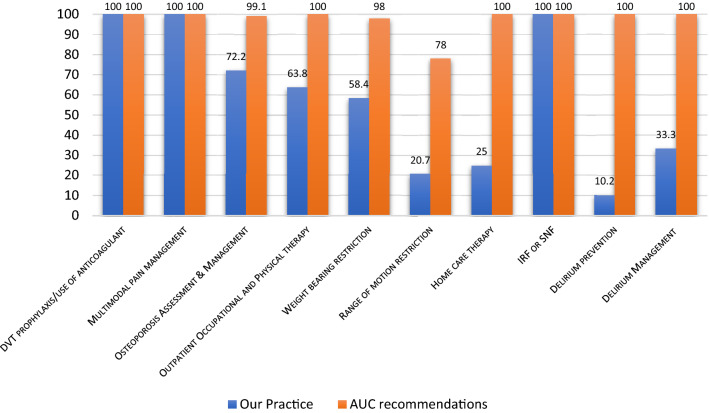
Appropriateness of our rehabilitation procedures and AUC recommendations

The actual treatment was appropriate and in agreement with the AUC recommendations in (100%) of three procedures (Deep venous thrombosis (DVT) prophylaxis, pain management, and Inpatient Rehabilitation Facility (IRF) or Skilled Nursing Facility (SNF)), in (72.2%) of osteoporosis and assessment management (*P* < 0.01), in (63.8%) of outpatient occupational and physical therapy (*P* = 0.01), in (25%) of home care therapy (*P* < 0.001), in (10.2%) of delirium prevention (*P* < 0.001), and in (33.3%) of delirium management (*P* = 0.25). Table 3 and Fig. 3 summarize the rate of appropriateness and the agreement with AUC recommendations.

**Table 3:** Actual rehabilitation procedures appropriateness and agreement with the AUC recommendations

Rehabilitation Types	Provided	Not provided	Removed	Appropriate	Maybe	Rarely	Agreementwith AUC	*p* value
DVT prophylaxis/use of anticoagulant	101	–	–	101 (100%)	–	–	100%	–
Multimodal pain Management	101	–	–	101 (100%)	–	–	100%	–
Osteoporosis Assessment & Management	73	28	–	73 (72.2%)	–	–	72.2%	< .01
Outpatient Occupational and Physical therapy	30	17	54	30 (63.8%)	–	–	63.8%	.01
Weight bearing restriction	59	42	–	–	1 (1%)	58 (57.4%)	58.4%	< .001
Range of motion restriction	21	80	–	–	18 (17.7%)	3 (3%)	20.7%	< .001
Home care therapy	13	39	49	13 (25%)	–	–	25%	< .001
IRF or SNF †	27	–	74	27 (100%)	–	–	100%	–
Delirium prevention	10	88	3	10 (10.2%)	–	–	10.2%	< .001
Delirium Management	1	2	98	1 (33.3%)	–	–	33.3%	.25

^†^ = Inpatient Rehabilitation or Skilled Nursing Facility

## Discussion

The most important finding of this study was that the appropriateness rate in our patients was significantly lower than the AUC recommendations for the appropriate and maybe appropriate treatments; however, the rarely appropriate treatment was not statistically different compared with the AUC recommendation.

Furthermore, this study demonstrated a remarkable variation in the appropriateness of post-operative rehabilitation procedures for elderly hip fractures at our center. It was appropriate and in agreement with AUC recommendations only in a few procedures and, to a lesser extent, in the other rehabilitation procedures.

It is concerning that some rehabilitation procedures were not provided in a high percentage of patients at our institution, like home care therapy, delirium prevention, delirium management. The reasons might be due to the lack of evidence-based post-operative rehabilitation protocols for hip fractures in elderly patients at our institute, underestimation of the importance of these procedures by some surgeons, and/or the lack of the orthogeriatric service at our center. Consequently, implementation of AUC for post-operative rehabilitation of hip fractures in elderly could allow surgeons to explore all available and evidence-based rehabilitation options. It is a known fact from the literature and AUC guidelines that the post-operative rehabilitation procedures should be provided for any elderly patients with hip fractures to improve the quality of patient care by guiding the treating physicians in selecting an appropriate rehabilitation procedure. Hence, the variation in the post-operative rehabilitation for elderly patients with hip fractures should be decreased, and the patient care should be improved [2, 3, 15, 16].

Several authors have investigated the importance of the evidence-based post-operative rehabilitation protocols, and the orthogeriatric settings for the management of the elderly hip fracture [18–22].

Mark et al. demonstrated that hip fractures should be treated according to the most up-to-date evidence to achieve the best possible outcomes and optimal use of limited resources [18].

Beaupre et al. reported that the implementation of an evidence-based clinical pathway reduced the post-operative morbidity and did not affect in-hospital mortality or overall costs of inpatient care after hip fractures [19].

Adunsky et al. reported that the functional outcome of elderly hip fracture patients is better for those treated in the orthogeriatric setting, as compared with the common two-steps model of orthopedic surgery followed by transfer to a geriatric rehabilitation facility [20].

Siddiqi et al. emphasized that a proactive geriatric consultation may reduce delirium incidence and severity in patients undergoing surgery for hip fracture [21].

We also found that the application of patients’ data into the free web-based AUC application made post-operative rehabilitation options for each case is relatively easy and simple to use. Most of the AUC rehabilitation therapies were provided at our center; however, not all the AUC scenarios were observed in our patients.

The availability of the AUC as a free web-based application is a valuable tool that can help the orthopedic surgeons to build their practice on a solid evidence-based data to improve the quality of care in such group of patients.

The AUC for the surgical treatment of knee osteoarthritis (OA) was recently evaluated, and concluded that AUC for the surgical treatment of knee OA can be applied easily in a clinical setting with 99% agreement was observed between the actual treatment of 100 patients and the AUC recommendations of appropriate treatment [22].

This study also has shown that the hip fractures were more common in male patients with a low prevalence of cognitive impairment and post-operative delirium.

The previous studies reported that the elderly hip fractures most commonly occurred in women with a higher prevalence of cognitive impairment and post-operative delirium [23–27].

These variations might be attributed to the health, cultural, and demographic variation across the countries and/or the global geographic variations in hip fractures and cognitive impairment [28, 29].

In Qatar, the population characteristics are male predominance with a ratio of 2.6:1 because it is a newly growing country and depends mainly on male laborers from other countries and constitutes about 72% of the total population [30]. Additionally, patients with cognitive problems and other disabling condition tend to go back to their home country as they will be unable to work. Furthermore, the cognitive impairment and post-operative delirium might be underestimated in the pre-operative and post-operative assessment of our patients.

There were some drawbacks of AAOS published AUC for PORHFE: The appropriateness was not clearly defined for each recommendation of AUC, such as what is the alternative to the appropriateness ratings if the recommended procedure was not provided. For example, the range of motion restriction and weight-bearing restriction was always graded as maybe or rarely appropriate in all AUC scenarios; however, they did not specify how it graded the appropriateness if there were no restrictions.

Sherrington et al. reported in a randomized clinical trial that weight-bearing and non-weight-bearing exercise programs produce similar effects on strength, balance, gait, and functional performance among inpatients after hip fracture [31].

Home care therapy is a treatment that still controversial, where not all patients or hospitals could afford such treatment after the AUC amendment.

## Limitations

This study has several limitations: the retrospective design of study, lack of comparative group, and patient outcome. The lack of sample size calculation, low level of evidence, and the cultural difference across the countries might affect the appropriateness ratings are other limitations of this study.

## Conclusions

This study demonstrated that there is a remarkable variation in the appropriateness of the various post-operative rehabilitation procedures for elderly hip fracture. Additionally, the AUC application was easy to use and simple for identifying post-operative rehabilitation protocols for elderly hip fractures in the trauma setting, hence, we recommend to use it in the clinical practice.

## Data Availability

The datasets generated during and/or analyzed during the current study are available from the corresponding author on reasonable request.
